# Relevance of long-lived CD8^+^ T effector memory cells for protective immunity elicited by heterologous prime-boost vaccination

**DOI:** 10.3389/fimmu.2012.00358

**Published:** 2012-12-04

**Authors:** José R. Vasconcelos, Mariana R. Dominguez, Adriano F. Araújo, Jonatan Ersching, Cibele A. Tararam, Oscar Bruna-Romero, Mauricio M. Rodrigues

**Affiliations:** ^1^Centro de Terapia Celular e Molecular, Universidade Federal de São Paulo - Escola Paulista de MedicinaSão Paulo, São Paulo, Brazil; ^2^Departamento de Microbiologia, Imunologia e Parasitologia, Universidade Federal de São Paulo - Escola Paulista de MedicinaSão Paulo, São Paulo, Brazil; ^3^Departamento de Microbiologia, Instituto de Ciências Biológicas, Universidade Federal de Minas GeraisBelo Horizonte, Minas Gerais, Brazil

**Keywords:** memory, vaccines, CD8, adenovirus

## Abstract

Owing to the importance of major histocompatibility complex class Ia-restricted CD8^+^ T cells for host survival following viral, bacterial, fungal, or parasitic infection, it has become largely accepted that these cells should be considered in the design of a new generation of vaccines. For the past 20 years, solid evidence has been provided that the heterologous prime-boost regimen achieves the best results in terms of induction of long-lived protective CD8^+^ T cells against a variety of experimental infections. Although this regimen has often been used experimentally, as is the case for many vaccines, the mechanism behind the efficacy of this vaccination regimen is still largely unknown. The main purpose of this review is to examine the characteristics of the protective CD8^+^ T cells generated by this vaccination regimen. Part of its efficacy certainly relies on the generation and maintenance of large numbers of specific lymphocytes. Other specific characteristics may also be important, and studies on this direction have only recently been initiated. So far, the characterization of these protective, long-lived T cell populations suggests that there is a high frequency of polyfunctional T cells; these cells cover a large breadth and display a T effector memory (TEM) phenotype. These TEM cells are capable of proliferating after an infectious challenge and are highly refractory to apoptosis due to a control of the expression of pro-apoptotic receptors such as CD95. Also, they do not undergo significant long-term immunological erosion. Understanding the mechanisms that control the generation and maintenance of the protective activity of these long-lived TEM cells will certainly provide important insights into the physiology of CD8^+^ T cells and pave the way for the design of new or improved vaccines.

## GENETIC VACCINATION USING THE HETEROLOGOUS PRIME-BOOST REGIMEN

Genetic vaccination using naked DNA or recombinant viruses is being pursued as an alternative to traditional vaccines. This strategy could be particularly important in the case of intracellular pathogens and neoplastic cells, where the effectiveness relies heavily on the capacity of the vaccine to elicit specific immune responses mediated by cytotoxic CD8^+^ T cells (reviewed in Rice et al., 2008; Lasaro and Ertl, 2009 Bassett et al., 2011; Gómez et al., 2011; Mudd and Watkins, 2011; Saade and Petrovsky, 2012).

Whereas the use of single-vector delivery for priming and boosting is usually the initial option, one of the most prolific areas of genetic vaccine development is the strategy known as the heterologous prime-boost regimen. This strategy uses two different vectors, each carrying a gene that encodes the same antigenic protein for priming and boosting immunizations. The utility and importance of this strategy was first proposed in the early 1990s using a combination of recombinant viruses (influenza and vaccinia) to induce protective immunity against malaria (Li et al., 1993; Rodrigues et al., 1994). Subsequently, this approach was extended and simplified by incorporating naked DNA for priming followed by a booster injection of a recombinant poxviral vector (i.e., modified vaccinia Ankara, MVA); this was also used to generate sterile protective immunity against rodent malaria (Schneider et al., 1998; Sedegah et al., 1998). Collectively, these studies demonstrated that the heterologous prime-boost regimen generated significantly higher immune responses and proved more effective than the use of any of these genetic vectors individually. The initial use of rodent malaria parasites as a model system may have delayed the development of the field, because malaria is not a conventional model system for developing antiviral or antibacterial vaccines. Nevertheless, this fortuitous fact established the important concept that the difficulty in generating immunity to malaria, and perhaps to other intracellular parasites, in many ways recapitulates the struggles encountered to elicit protective immunity against viruses and bacteria that case chronic infections such as HIV and tuberculosis (TB).

In subsequent years, the heterologous prime-boost vaccination regimen was adopted worldwide as a powerful means to elicit strong type 1 effector CD8^+^ T cell-mediated immune responses (Tc1) against viral, parasitic, and neoplastic antigens in rodents and non-human primates (NHP; Irvine et al., 1997; Amara et al., 2001; McShane et al., 2001; Zavala et al., 2001; Moore and Hill, 2004; Ellenberger et al., 2006; Gilbert et al., 2006; Aidoo et al., 2007; Nigam et al., 2007; Martinon et al., 2008; Sadagopal et al., 2008). Based on pre-clinical studies, a number of human clinical trials have also been initiated. However, to our knowledge, heterologous prime-boost regimens using plasmid DNA and recombinant poxviruses have not yet provided meaningful protective immunity in humans (McConkey et al., 2003; Moorthy et al., 2004; Keating et al., 2005; Vuola et al., 2005; Cebere et al., 2006; Dunachie et al., 2006; Goonetilleke et al., 2006). The precise reason for such failures is not yet clear. It may be due to the target antigens chosen or to the possibility that the combination of vectors may elicit a type of effector CD8^+^ T cells in humans that are not functionally and/or phenotypically related to mice, as discussed below.

A number of possible vector combinations that significantly improved cell-mediated immunity, particularly the generation of specific CD8^+^ T cells, have been described in parallel. Among them, heterologous prime-boost vaccination using naked plasmid DNA for priming followed by a booster injection of recombinant replication-deficient human adenovirus 5 (AdHu5) has recently received significant attention. This strategy has proved successful in some relevant experimental models such as simian immunodeficiency virus (SIV), malaria, Marburg, and Ebola virus infection, and Chagas disease (American trypanosomiasis), providing a considerable degree of protective immunity (Gilbert et al., 2002; Casimiro et al., 2003, 2005; Santra et al., 2005; Acierno et al., 2006; Letvin et al., 2006; Mattapallil et al., 2006; Sun et al., 2006; Wilson et al., 2006; de Alencar et al., 2009; Geisbert et al., 2010; Hensley et al., 2010; Martins et al., 2010; Dominguez et al., 2011; Rigato et al., 2011). These relative successes obtained in pre-clinical experimental models fueled human phase I trials (Freel et al., 2010; Jaoko et al., 2010; Koup et al., 2010; Schooley et al., 2010; Churchyard et al., 2011; De Rosa et al., 2011).

Very recently, improved vector combinations have yielded results (measured in terms of protective immunity) that are slightly better than the results obtained by using plasmid DNA followed by replication-deficient AdHu5 viruses. These new strategies include (i) prime with plasmid DNA in the presence of cytokine genes such as IL-12 or GM-CSF (Lai et al., 2011; Winstone et al., 2011), (ii) genes encoding multimeric proteins (Lakhashe et al., 2011), (iii) boost of adenovirus-immunized animals with an optimized plasmid DNA (Hutnick et al., 2012), (iv) prime with rhesus cytomegalovirus (Hansen et al., 2011), and (v) prime with a different heterologous strain of adenovirus (Barouch et al., 2012).

The precise reason for the superior performance of the heterologous prime-boost vaccination compared to the sequential use of the same vector is still a matter of controversy. Some evidence indicates the possibility that the intense immunity to epitopes present on the priming vector prevents the boosting effect. For example, a recent study in humans shows that a second dose of a recombinant AdHu5 does not provide significant boosting. In parallel, recombinant AdHu5 boosting of DNA-primed individuals resulted in significantly higher immune responses (De Rosa et al., 2011). The anti-vector immunity can be either antibody mediated or independent (Cockburn et al., 2008; Schirmbeck et al., 2008; Frahm et al., 2012). Haut et al. (2011) found that in B cell-deficient mice transgene-specific CD8^+^ T cell responses were significantly higher in systemic compartments. In contrast, recent studies in humans showed that neutralizing antibodies titers to AdHu5 did not correlate with the magnitude of specific CD8^+^ T cell of priming after immunization with a recombinant AdHu5. In these experiments, the frequency of specific CD4^+^ T cells negatively correlated with the intensity of specific CD8^+^ T cells priming (Frahm et al., 2012).

In spite of the clear evidences that pre-existing immunity may interfere with the use of viral vectors, still, the heterologous prime-boost regimen of immunization is described as a possible solution to this problem. This can be achieved by strong priming with cytokine genes (for example, see Barouch et al., 2003).

Independent of the reasons why the heterologous prime-boost vaccination regimen is superior to the sequential use of the same vector, the main purpose of this review is to examine the characteristics of the protective CD8^+^ T cells generated by this vaccination regimen.

## CHARACTERISTICS OF PROTECTIVE CD8^+^ T CELLS ELICITED AFTER HETEROLOGOUS PRIME-BOOST VACCINATION

### HIGH FREQUENCIES OF SPECIFIC CD8^+^ T CELLS

One hallmark of the heterologous prime-boost regimen is the elicitation of a higher frequency of epitope-specific CD8^+^ T cells across multiple models. This high number of effector T cells was initially estimated by the presence of epitope-specific IFN-γ-producing cells using the *ex vivo* ELISPOT assay (Murata et al., 1996; Schneider et al., 1998; Sedegah et al., 1998; Bru n, 2001). Subsequently, the hypothesis was further validated by intracellular staining for IFN-γ (Pinto et al., 2003) and tetramer staining of epitope-specific CD8^+^ T cells (Tao et al., 2005). More recently, intracellular staining for TNF, IL-2, MIP1-β, T cell surface mobilization of CD107a, and *in vivo* cytotoxicity provided extended evidence (for examples, see Masopust et al., 2006; Mattapallil et al., 2006; Cox et al., 2008; de Alencar et al., 2009; Freel et al., 2010; Reyes-Sandoval et al., 2010; Rigato et al., 2011).

Because most studies are performed with T cells collected from the spleen or peripheral blood lymphocytes (PBL) of mice or NHP, respectively, it is not clear whether these results reflect an overall increase in every tissue. The presence of a large number of epitope-specific T cells in several tissues has been documented in the case of mouse lung, liver, intraepithelial lymphocytes, and PBL (Masopust et al., 2006; Reyes-Sandoval et al., 2011); however, because parallel comparison was not performed with animals that were immunized with a single vector, it is not clear whether these levels were particularly higher than the other vaccination protocols. Conversely, the frequency of epitope-specific CD8^+^ T cells seems to decrease in mouse lymph nodes (Masopust et al., 2006). This may be due to the lack of CD62L expression on the surface of these activated T cells, as discussed below. In addition, the pattern of circulation and recirculation of these lymphocytes has been poorly explored. A single study, however, demonstrated that, after a recombinant plasmid DNA prime-AdHu5 boost, CD8^+^ T cells need to recirculate in order to exert protective immunity against an infectious challenge with the protozoan parasite *Trypanosoma cruzi*. In these vaccinated mice, treatment with the drug FTY720 significantly reduced the efficacy of the protective immunity by interfering specifically with signaling through sphingosine-1-phosphate receptor-1, thereby inhibiting the egress of T cells from the lymph nodes (Dominguez et al., 2012). This observation is important because immunity to certain pathogens is not dependent on the recirculation of T lymphocytes (Pinschewer et al., 2000; Kursar et al., 2008; Jiang et al., 2012).

The importance of recirculation is likely dependent on factors such as (i) the host, (ii) the vectors used for prime and/or boost, and (iii) the route of administration of each vector (for examples, see Mattapallil et al., 2006; Kaufman et al., 2010). These are important characteristics that will certainly influence the protective immunity, as pathogens can cause either tissue-specific or systemic infections.

The T cell protective immunity elicited by heterologous prime-boost regimen is not only significantly higher than the immunity elicited by the traditional vaccine regimen but is also longer-lived. Several experimental models have shown that the immunity lasts for significant periods of time (Amara et al., 2001; Bru n, 2001; de Alencar et al., 2009; Reyes-Sandoval et al., 2010; Rigato et al., 2011).

### POLYFUNCTIONALITY OF SPECIFIC CD8^+^ T CELLS

Based on the assays described above, it became clear that the specific CD8^+^ T cells elicited by the heterologous prime-boost regimen could exert different immunological functions, as measured by *ex vivo* or *in vivo* assays. Confirmation of the polyfunctionality of these cells was made possible through FACS analyses coupling intracellular cytokine staining and cell surface mobilization of the degranulation marker CD107a.

Accordingly, a number of studies confirmed that distinct heterologous prime-boost regimens elicited polyfunctional CD8^+^ T cells as defined by the cells’ capability to exert two or more functions at the same time. The most frequent example of this across different models is specific CD8^+^ T cells producing IFN-γ and TNF simultaneously. High frequencies of polyfunctional specific CD8^+^ T cells were described in (i) mice (Masopust et al., 2006; Duke et al., 2007; de Alencar et al., 2009; Elvang et al., 2009; Reyes-Sandoval et al., 2010; Rigato et al., 2011; Rodríguez et al., 2012; Vijayan et al., 2012), (ii) NHP (Mattapallil et al., 2006; Sun et al., 2006; Cox et al., 2008; Liu et al., 2008; Magalhaes et al., 2008; Cayabyab et al., 2009; Wilks et al., 2010; Hutnick et al., 2012), and (iii) humans (Beveridge et al., 2007; Harari et al., 2008; Winstone et al., 2009; Freel et al., 2010; Jaoko et al., 2010; Koup et al., 2010; Schooley et al., 2010; Churchyard et al., 2011; De Rosa et al., 2011).

It is noteworthy that although these T cells have a high frequency of polyfunctionality, their ability to mediate multiple immunological functions has never been clearly linked to their protective capacity. It is possible that this characteristic aids in but is not critical for their effector functions, depending on the mechanism necessary for pathogen elimination. In a single study performed using genetically deficient mice, in the absence of either IFN-γ or perforin, heterologous prime-boost vaccination failed to mediate protective immunity against infection with the intracellular parasite *T. cruzi*. In the case of perforin-deficient mice, the lack of protective immunity was associated mainly with a significant decrease in the induction of polyfunctional T cells (de Alencar et al., 2009). A second study correlated the presence of higher frequencies of polyfunctional T cells to protective immunity against liver stages of malaria parasite (Reyes-Sandoval et al., 2010). Although these studies may suggest a role for polyfunctional T cells during protective immunity, it is still too early to conclude that they are critical for the protective immunity exerted by CD8^+^ T cells elicited following the heterologous prime-boost vaccination regimen.

### BREADTH OF SPECIFIC CD8^+^ T CELLS

T cell immune responses are often restricted to a few immunodominant epitopes, a phenomenon termed immunodominance (Akram and Inman, 2012). The precise reason for such a restriction is not clear; however, it may have evolved to maximize the immune response, while at the same time reducing the risk of autoimmunity. For the purpose of vaccine development, having only a narrow number of recognized epitopes may be dangerous, as the pathogens will rapidly select for escape mutants to avoid effector immune responses (reviewed in Streeck and Nixon, 2010; Chopera et al., 2011).

Although it has been possible to increase the frequency of T cells specific for immunodominant epitopes, it is still a challenge to broaden the vaccine-induced CD8^+^ T cell response to a number of subdominant T cell epitopes. There is evidence that heterologous rAdHu5 boosting improved not only the magnitude but also the breadth of specific CD8^+^ cells (Liu et al., 2008). However, the impact of this response on protective immunity is not clear. Two recent studies indicated that immunity to subdominant epitopes might participate in vaccine-induced protective immunity following a DNA prime-AdHu5 boost vaccination regimen. The first study provided a correlation between the breadth of the immune response and the protective immunity observed in individual rhesus monkeys vaccinated with SIV genes (Martins et al., 2010). A second study formally demonstrated that heterologous prime-boost vaccination with plasmid DNA followed by recombinant AdHu5 elicited strong immune response to two subdominant epitopes that were not recognized during infection (Dominguez et al., 2011). Based on these observations, mutant genes were generated in which the dominant epitope was removed. Heterologous prime-boost vaccination with these mutant genes-induced CD8^+^ T cell immune responses only to the subdominant epitopes. Most importantly, strong CD8^+^ T cell-mediated immunity was still observed (Dominguez et al., 2011). These results unequivocally demonstrate the importance of immunity to the subdominant epitopes and the ability of the heterologous prime-boost to elicit them. Nevertheless, other groups still have difficulty improving the immune response to subdominant epitopes following heterologous prime-boost vaccination of NHP (Vojnov et al., 2011). Therefore, new strategies to improve the breadth of the immune response might be developed in order to potentiate vaccine formulation.

### TEM PHENOTYPE OF SPECIFIC LONG-LIVED CD8^+^ T CELLS

The current immunological paradigm divides antigen-experienced CD8^+^ T cells into three main types: (i) T effector (TE), (ii) T effector memory (TEM), and (iii) T central memory (TCM). These populations of T cells can be distinguished by the presence of activation markers, as well as by differences observed in their localization and recirculation patterns and their ability to proliferate and present certain effector functions/molecules (Angelosanto and Wherry, 2010; Cui and Kaech, 2010; Ahmed and Akondy, 2011; Sheridan and Lefrançois, 2011). More recently, other subpopulations of CD8^+^ T cells have also been described (Wakim et al., 2010; Sheridan and Lefrançois, 2011; Jiang et al., 2012). The concept of TE/TEM/TCM was primarily established by experimental infections with self-curing pathogens. In these cases, TE are short-lived effector cells (CD44^High^ CD11a^High^ CD62L^Low^ CD127^Low^ KLRG1^High^); TCM are long-lived memory cells (CD44^High^ CD11a^High^ CD62L^High^ CD127^High^ KLRG1^High^); and TEM are transitional cells that exist for a short period of time and have distinct surface marker expression (CD44^High^ CD11a^High^ CD62L^Low^ CD127^High^ KLRG1^High^). However, even by using models of self-curing infections, the existence of other memory T cell subpopulations that might be more rele-vant for long-term T cell immunity have been proposed (Hikono et al., 2007).

In addition, very recently, considerable interest has been placed on tissue-resident memory T (TRM) cells. These cells are potent long-lived effector cells present in a variety of peripheral tissues including the skin and sensory ganglia, gut, brain, lung, etc. (Wakim et al., 2008; Gebhardt et al., 2009; Masopust et al., 2010; Purwar et al., 2011). They mediate protective immunity against brain infection with vesicular stomatitis virus or skin infection with vaccinia or herpes simplex virus (Wakim et al., 2010; Jiang et al., 2012; Mackay et al., 2012). Their phenotype is CD44^High^, CD62L^Low^, CD103^High^, CD69^High^, CD127^Low^, and PD-1^Low^. The relationship of TRM cells with TEM or TCM is yet to be determin-ed. Some authors propose that these cells constitute an independ-ent lineage primed within the tissue (Wakim et al., 2010, 2012).

Very recently, the development of a methodology to perform transcriptional profiling at the single cell level has detected further differentiation profiles among TEM and TCM. The analysis of specific splenic CD8^+^ T cells from mice immunized with distinct vaccination protocols yields significant differences among these subpopulations (Flatz et al., 2011). For example, while 74% of the specific TEM elicited by the heterologous DNA prime-recombinant AdHu5 boost vaccination were Klrk1^–^ Klrg1^–^ Ccr5^–^, only 20% of the cells generated by HuAd5-prime-recombinant LCMV boost had a similar phenotype (Flatz et al., 2011). This type of analysis further highlights the heterogeneity still being uncovered within these memory cells.

It has been observed on multiple occasions that after heterologous prime-boost vaccination, the boosting immunization drives not only an expansion of the T cells but also a different phenotype in the long-lived memory T cells. Different doses of the vaccine also caused a significant increase in the frequency of specific CD8^+^ T cells with a TEM phenotype (CD44^High^ CD11a^High^ CD62L^Low^ CD127^High^ KLRG1^High^; Masopust et al., 2006). TEM cells were described initially as highly protective against certain viral and bacterial infections (Bachmann et al., 2005a,b; Huster et al., 2006). Likewise, protective immunity afforded by the different heterologous prime-boost vaccination protocols has been associated with the presence of this type of T cell (Hansen et al., 2011; Reyes-Sandoval et al., 2011; Rigato et al., 2011; Xiao et al., 2011; Barouch et al., 2012; Yamamoto et al., 2012).

**FIGURE 1:**
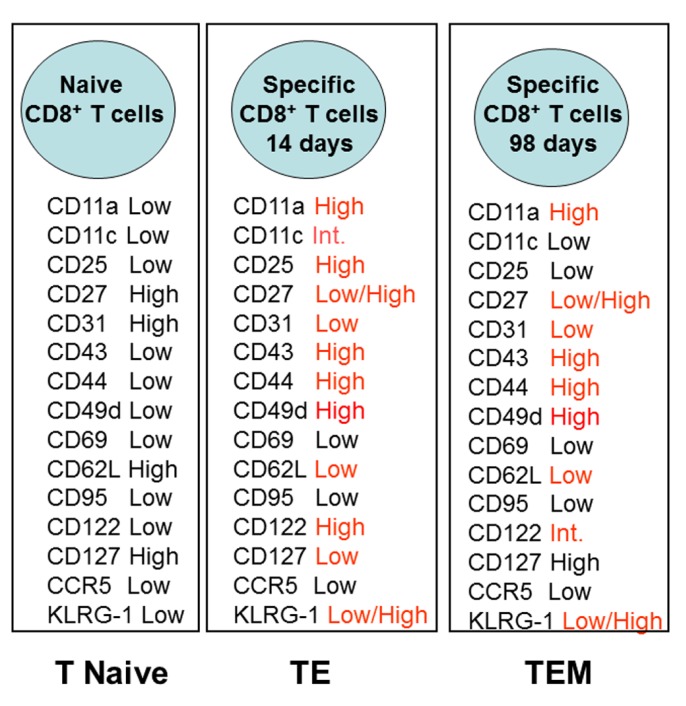
**Phenotype of specific CD8^+^ T cells elicited by heterologous prime-boost vaccination using recombinant plasmid DNA and AdHu5**. Prime-boost regimen was performed as detailed described by Rigato et al. (2011). Mice were primed i.m. with plasmid DNA (100 µg) and boosted 21 days later with AdHu5 (2 × 10^8^) both expressing the gene encoding the amastigote surface protein-2 of *T. cruzi*. Expression of distinct adhesion/activation receptors on the surface of splenic CD8^+^ specific T cells is shown at day 14 or day 98 after the boost immunization.

Based on the relatively poor knowledge of the surface activation markers present on long-lived specific TEM CD8^+^ T cells, we performed a detailed analysis of the different T cells markers following intramuscular DNA prime-adenovirus boost immunization. We identified transgenic epitope-specific T cells in the spleen of immunized mice 14 or 98 days after the boost vaccination. **Figure 1** summarizes the surface marker phenotype of these epitope-specific T cells compared to the phenotype of the naïve cells.

### PROLIFERATIVE CAPACITY OF SPECIFIC CD8^+^ T CELLS

The proliferative capacity of specific T cells elicited by heterologous prime-boost vaccination has not been thoroughly studied to date. In general, after resolution of experimental self-curing infections, the frequency of total CD8^+^ T cells declines to less than 10% of the maximal number of specific T cells observed during the peak of the immune response; this is known as the contraction phase. The decrease in the number of specific T cells occurs mainly among the short-lived effector cells (Angelosanto and Wherry, 2010; Cui and Kaech, 2010; Ahmed and Akondy, 2011; Sheridan and Lefrançois, 2011). However, this does not seem to be the case for the TE cells elicited by the heterologous prime-boost vaccination regimen (Jameson and Masopust, 2009). In this case, the cells expand and are maintained at a high frequency in the spleen for a long period of time (Masopust et al., 2006; Vezys et al., 2009; Rigato et al., 2011). This is a somewhat unexpected finding and might be of great relevance for the success of this vaccination protocol. Because this expansion and maintenance contrasts with the retraction observed after self-curing infections, it opens a number of questions that should be studied in depth: (i) How can cells expand out of control to reach frequencies higher than the primary immune response? (ii) How do T cells avoid the apoptotic process observed for the short-lived effector cells during the contraction phase? (iii) How are T cells maintained for these long periods? These questions are keys to understanding the physiology of CD8^+^ T cells and developing better T cell vaccines.

After an infectious challenge, specific T cells proliferate. However, it is still unknown whether this proliferative response occurs among the antigen-primed TE(M) cells, rare TCM or naïve T cells. To address this question, it is now possible to use the gzmBCreERT2/ROSA26EYFP transgenic mouse line (Bannard et al., 2009). Specific TE CD8^+^ T lymphocytes can be indelibly labeled with enhanced yellow fluorescent protein (EYFP). Following an infectious challenge, it will be possible to follow the expansion of EYFP-labeled TE cells. This result will confirm or not whether the cells that expand after the infectious challenge are indeed mainly antigen-experienced TE. Also, by the adoptive transference of green-fluorescent naïve specific T cells prior to the infectious challenge it will be possible to determine whether these cells proliferate or not together with the specific TE cells. Whether this is true for all different protocols of heterologous prime-boost vaccination strategies is still unknown and might be relevant for the development of vaccines to be used in individuals primed with conventional vaccines such as Bacillus Calmette–Guérin (BCG; Hoft et al., 2012).

### REFRACTORINESS TO APOPTOSIS OF SPECIFIC CD8^+^ T CELLS

As mentioned above, after an intense immune response and pathogen elimination, the number of specific CD8^+^ T cell is drastically reduced during a period called the contraction phase. In that period, TE or short-lived effector cells are eliminated by mechanisms that involve apoptosis mediated by BCL-2-interacting mediator of cell death (Bim) and CD95 (also known as FAS; Green, 2008; Hughes et al., 2008; Weant et al., 2008; Bouillet and O’Reilly, 2009). We observed that following prime-boost immunization with recombinant plasmid DNA and AdHu5, TEM cells do not upregulate surface CD95 expression and are refractory to anti-CD95-induced apoptosis *in vitro* (Rigato et al., 2011). Because CD95 is an important initiator of the intrinsic pathway of apoptosis in T lymphocytes, low levels of expression may protect T cells from apoptotic death.

Decreased levels of expression or resistance to activation of other receptors that control T cell death, such as TNF receptor and TRAIL, might also play a role during the survival of specific TEM cells. In contrast, increased expression of other receptors may act to protect these specific T cells. Recently, expression of PDL-1 (B7-H1) on antigen-activated CD8^+^ T cells made these cells more resistant to Ca^2^^+^-dependent and Fas ligand-dependent killing by cytotoxic T lymphocytes (Pulko et al., 2011). However, more details are necessary to understand the expression levels of many of these molecules controlling T cell survival.

It is plausible that Bim is poorly activated, or not activated at all, on these cells. Because Bim is activated in response to the lack of certain external stimuli, these activation signals might be maintained for long-term. One such mechanism is antigen presentation by dendritic cells. Using a replication-deficient adenoviral vector, Tatsis et al. (2007) demonstrated that the transgene product remains available for antigen presentation for as long as 16 weeks after a single immunizing dose. The importance of continuous transgene expression in the maintenance of the specific CD8^+^ T cells after AdHu5 vaccination was also demonstrated by a causal relationship between both using an inducible system to turn off the transgene expression (Finn et al., 2009). Bassett et al. (2011) showed that both hematopoietic and non-hematopoietic antigen-presenting cells are necessary for the maintenance of CD8^+^ T cells following immunization with recombinant adenovirus. However, it is not clear whether antigen persistence is also observed with other vectors.

We tested whether the IFN-γ or IL12/IL23 signaling pathways might be important for maintaining these long-lived CD8^+^ TEM cells. The absence of either pathway individually made little difference in the generation of specific CD8^+^ T cells. The absence of the IL12/IL23 pathway, but not the IFN-γ pathway, was important for the long-term survival of these cells (Rigato et al., 2011). Further details regarding the relevance of these signaling pathways in maintaining a high frequency of CD8^+^ T cells are unknown. In addition, little is known about the impact of the lack of the IL12/IL23 pathway in the maintenance of specific CD8^+^ T cells after other heterologous prime-boost vaccination regimens. We consider this area of critical importance in understanding how T cells can be maintained at high frequencies for long periods of time. A possible explanation that remains to be tested is whether the lack of contraction could be due to the fact that many specific long-lived CD8^+^ T cells express low levels of the chemokine receptors CXCR3 and CCR5 (Flatz et al., 2011). This possibility is supported by recent observations that genetically deficient CD8^+^ T cells that do not express both these receptors were refractory to contraction and accumulated in higher numbers in the spleen (Kohlmeier et al., 2011).

**FIGURE 2:**
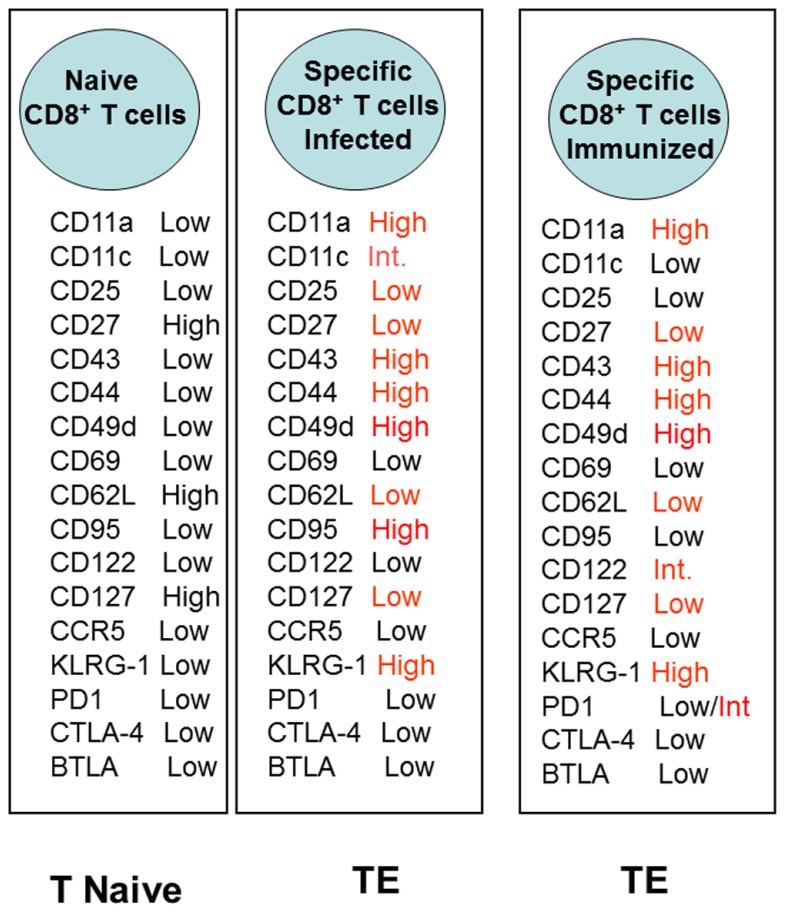
**Expression of distinct adhesion/activation receptors on the surface of specific CD8^+^ T cells elicited by either *T. cruzi* infection or recombinant AdHu5 immunization (Vasconcelos et al., 2012**). Mice were infected s.c. with *T. cruzi* (150 blood stream trypomastigotes) or immunized i.m. with AdHu5 (2 × 10^8^ pfu) expressing the gene encoding the amastigote surface protein-2 of *T. cruzi* 19 days earlier.

Recently, we described a new and potentially very important aspect of CD8^+^ TE cells. After recombinant AdHu5 vaccination, CD8^+^ TE cells do not undergo apoptosis, as well as prevent the development of a pro-apoptotic phenotype that occurs during experimental infection with the protozoan parasite *T. cruzi*. This phenomenon was observed when the administration of the recombinant AdHu5 vaccine was provided before (as part of a prime-boost regimen or alone) or at the time of the infectious challenge. AdHu5 treatment modulated specifically the CD8^+^ TE cells to express lower levels of CD95 (FAS) and become resistant to CD95-induced apoptosis. The determination of the distinct adhesion/activation receptors on the surface of the CD8^+^-specific T cells elicited by either infection or recombinant AdHu5 immunization showed very limited differences that were almost exclusively confined to the apoptotic receptor CD95 (**Figure 2**).

**FIGURE 3:**
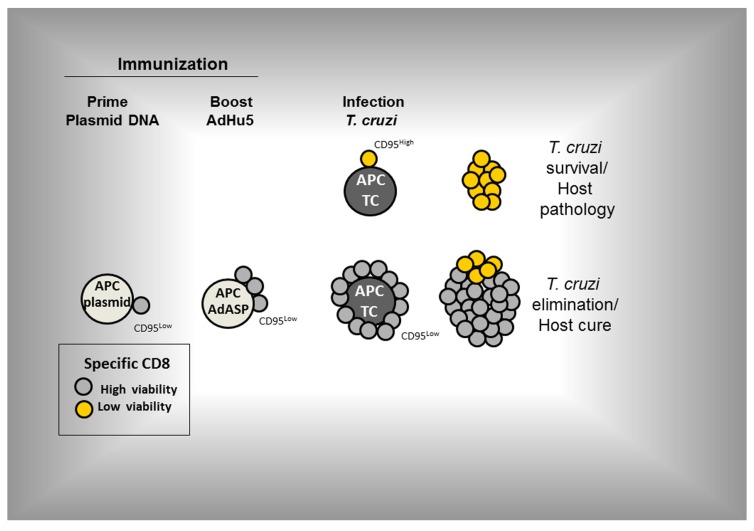
**The proposed pathway of activation of specific CD8^+^ T cells following prime-boost vaccination and infection (based on Vasconcelos et al., 2012)**. Prime-boost regimen was performed as detailed described by Rigato et al. (2011). Mice were primed i.m. with plasmid DNA (100 µg) and boosted 21 days later with AdHu5 (2 × 10^8^) both expressing the gene encoding the amastigote surface protein-2 of *T. cruzi*. Mice were infected s.c. with *T. cruzi* (150 blood stream trypomastigotes).

Despite the above results, we have not ruled out that other pro-apoptotic signaling pathways might also be altered. In these immunized and challenged mice, the CD8^+^ T cell population expanded largely and protected mice against an otherwise lethal infection (Vasconcelos et al., 2012). An important mechanism that controls the expression of CD95 on the surface of specific CD8^+^ T cells has just been described during development of immune responses to viral infections. TCR activation led to an increase of the RIG-I-like receptor LGP2 expression which down-regulated CD95 expression. In KO mice, the absence of this molecule significantly increased the apoptosis reducing the effectiveness of the immune response and resistance to the viral infection (Suthar et al., 2012). We consider this a promising area of study because it suggests that perhaps the control of T cell survival might be the key element for the development of new or improved vaccines. The proposed pathway of specific CD8^+^ T cell activation following prime-boost vaccination and infection is shown in **Figure 3** (based on Vasconcelos et al., 2012).

### REDUCED IMMUNOLOGICAL EROSION OF SPECIFIC CD8^+^ T CELLS

Memory CD8^+^ T cells are not a stable pool; subsequent infections may cause attrition and erosion of the memory T cell pool (Selin et al., 1996, 1999; Dudani et al., 2008; Huster et al., 2009; Schmidt and Harty, 2011). However, different heterologous prime-boost regimens generated a pool of CD8^+^ T cells that was not eroded by subsequent viral infections (Vezys et al., 2009; Rigato et al., 2011). After a vaccination regimen consisting of recombinant plasmid DNA prime-AdHu5 boost, we observed that viral infections had limited impact on the number or quality of the TEM cells as measured by different functional immunological assays. Most importantly, protective immunity mediated by these CD8^+^ TEM cells was not altered in these mice (Rigato et al., 2011).

In contrast, using a different prime-boost vaccination protocol consisting of dendritic cells coated with circumsporozoite protein peptide and a booster immunization with recombinant *actA*–*inlB*-deficient *Listeria monocytogenes* expressing the same epitope elicited an immune response that could be eroded by multiple subsequent infections (Schmidt and Harty, 2011). The discrepant results highlight the importance of determining the characteristic of each TEM elicited by the distinct regimen of vaccination, as suggested earlier (Flatz et al., 2011).

The resistance to immunological erosion is one more interesting characteristic of these long-lived TEM cells that has been poorly explored and may have an important impact in the development of efficient vaccines. As mentioned above, this high-resistance immunological erosion can be linked to the differential expression of surface molecules (death receptors such as CD95/FAS) or apoptosis mediators (such as Bim).

### UNKNOWN FATE OF THE SPECIFIC CD8^+^ TEM CELLS

Although some studies described that theses TEM can be long-lived in some mouse models, it is not clear what will be the fate of these cells on extended periods of time. Will they become TCM by upregulating CD62L? Will they simple die? Can they be boosted? Perhaps these questions can be addressed by using the gzmBCreERT2/ROSA26EYFP transgenic mouse line (Bannard et al., 2009). Labeling specific TE CD8^+^ T lymphocytes will allow us to follow theses cells for longer periods of time.

Also, their long-term fate in NHP or humans is even more important for the purpose of development of practical vaccines. Due to the obvious constrains, few studies so far have addressed this issue.

## CONCLUDING REMARKS AND PERSPECTIVES

Genetic vaccination using heterologous prime-boost regimen protocols has the potential to serve as the basis for the development of new vaccines against many pathogens. In spite of the progress over the past 20 years, much work is required to improve the relevance of vaccine research, considering that human immune response is still at least one order of magnitude lower than that observed in mouse or even NHP models.

Several areas can be pursued for this matter. So far, protective immunity is clearly associated with the presence of a high number of long-lived specific polyfunctional TEM cells. Nevertheless, several points should be considered. First, TEM may vary from one protocol to the other. It is also important to improve the number of specific TCM cells, for example, by using pharmacological modulators (Li et al., 2012; Takai et al., 2012). However, it will be a challenge to increase the number of TCM cells without reducing the number of TEM cells. Fine tuning the TEM subpopulations and increasing the number of TCM cells could further improve and prolong protective immunity beyond the levels currently achieved. Furthermore, new strategies to broaden the epitopes recognized by protective T cells would also improve both the quantity and the quality of the immune response. Finally, the circulation of these cells should be further studied for the purpose of personalizing the vaccination regimen for different types of infections, considering that the site of entry of each pathogen will vary, as will the localization of the T cells at the time of the infection.

In summary, the study of the control of memory T cell generation, maintenance, quality, and recirculation after distinct heterologous prime-boost vaccination regimens will provide important clues regarding the physiology of lymphocytes and the immune system, with potential applications in public health.

## Conflict of Interest Statement

Mauricio M. Rodrigues and Oscar Bruna Romero are named inventors on patent applications covering Trypanosoma cruzi vectored vaccines and immunization regimens. The other authors declare that the research was conducted in the absence of any commercial or financial relationships that could be construed as a potential conflict of interest.

## Acknowledgments

This work was supported by grants from Fundação de Amparo à Pesquisa do Estado de São Paulo (2009/06820-4), Instituto Nacional de Ciência e Tecnologia em Vacinas (INCTV-CNPq), The Millennium Institute for Vaccine Development and Technology (CNPq - 420067/2005-1), The Millennium Institute for Gene Therapy (Brazil), Malaria-PRONEX and PNPD. JoséR. Vasconcelos, Adriano F. Araújo, Mariana R. Dominguez, and Cibele A. Tararam are recipients of fellowship from FAPESP. JoséR. Vasconcelos, Oscar Bruna-Romero, and Mauricio M. Rodrigues are recipients of fellowships from CNPq.

## References

[B1] Acierno P. M. Schmitz J. E. Gorgone D. A. Sun Y. Santra S. Seaman M. S. (2006). Preservation of functional virus-specific memory CD8+ T lymphocytes in vaccinated, simian human immunodeficiency virus-infected rhesus monkeys. *J. Immunol.* 176 5338–5345 16622001 10.4049/jimmunol.176.9.5338

[B2] Ahmed R. Akondy R. S. (2011). Insights into human CD8+ cell memory using the yellow fever and smallpox vaccines. *Immunol. Cell Biol.* 89 340–345 21301482 10.1038/icb.2010.155

[B3] Aidoo M. Otten R. A. Rodriguez V. Sariol C. A. Martinez M. Kraiselburd E. (2007). Absence of SHIV infection in gut and lymph node tissues in rhesus monkeys after repeated rectal challenges following HIV-1 DNA/MVA immunizations. *Vaccine* 25 6474–6481 17688978 10.1016/j.vaccine.2007.06.014

[B4] Akram A. Inman R. D. (2012). Immunodominance: a pivotal principle in host response to viral infections. *Clin. Immunol.* 143 99–115 22391152 10.1016/j.clim.2012.01.015

[B5] Amara R. R. Villinger F. Altman J. D. Lydy S. L. O’Neil S. P. Staprans S. L. (2001). Control of a mucosal challenge and prevention of AIDS by a multiprotein DNA/MVA vaccine. *Science* 292 69–74 11393868 10.1126/science.1058915

[B6] Angelosanto J. M. Wherry E. J. (2010). Transcription factor regulation of CD8+ T-cell memory and exhaustion. *Immunol. Rev.* 236 167–175 20636816 10.1111/j.1600-065X.2010.00927.x

[B7] Bachmann M. F. Wolint P. Schwarz K. Jäger P. Oxenius A. (2005a). Functional properties and lineage relationship of CD8+ T cell subsets identified by expression of IL-7 receptor alpha and CD62L. *J. Immunol.* 175 4686–4696 16177116 10.4049/jimmunol.175.7.4686

[B8] Bachmann M. F. Wolint P. Schwarz K. Oxenius A. (2005b). Recall proliferation potential of memory CD8+ T cells and antiviral protection. *J. Immunol.* 175 4677–4685 16177115 10.4049/jimmunol.175.7.4677

[B9] Bannard O. Kraman M. Fearon D. T. (2009). Secondary replicative function of CD8+ T cells that had developed an effector phenotype. *Science* 323 505–509 19164749 10.1126/science.1166831 PMC2653633

[B10] Barouch D. H. (2010). Novel adenovirus vector-based vaccines for HIV-1. *Curr. Opin. HIV AIDS* 5 386–390 20978378 10.1097/COH.0b013e32833cfe4c PMC2967414

[B11] Barouch D. H. Liu J. Li H. Maxfield L. F. Abbink P. Lynch D. M. (2012). Vaccine protection against acquisition of neutralization-resistant SIV challenges in rhesus monkeys. *Nature* 7383 89–93 22217938 10.1038/nature10766 PMC3271177

[B12] Barouch D. H. McKay P. F. Sumida S. M. Santra S. Jackson S. S. Gorgone D. A. (2003). Plasmid chemokines and colony-stimulating factors enhance the immunogenicity of DNA priming-viral vector boosting human immunodeficiency virus type 1 vaccines. *J. Virol.* 77 8729–8735 12885892 10.1128/JVI.77.16.8729-8735.2003 PMC167238

[B13] Bassett J. D. Yang T. C. Bernard D. Millar J. B. Swift S. L. McGray A. J. (2011). CD8+ T-cell expansion and maintenance after recombinant adenovirus immunization rely upon cooperation between hematopoietic and nonhematopoietic antigen-presenting cells. *Blood* 117 1146–1155 21088134 10.1182/blood-2010-03-272336

[B14] Beveridge N. E. Price D. A. Casazza J. P. Pathan A. A. Sander C. R. Asher T. E. (2007). Immunisation with BCG and recombinant MVA85A induces long-lasting, polyfunctional *Mycobacterium tuberculosis*-specific CD4+ memory T lymphocyte populations. *Eur. J. Immunol.* 37 3089–3100 17948267 10.1002/eji.200737504 PMC2365909

[B15] Bouillet P O’Reilly L. A. (2009). CD95, BIM and T cell homeostasis. *Nat. Rev. Immunol.* 9 514–519 19543226 10.1038/nri2570

[B16] Bruña-Romero O. González-Aseguinolaza G. Hafalla J. C. Tsuji M. Nussenzweig R. S. (2001). Complete, long-lasting protection against malaria of mice primed and boosted with two distinct viral vectors expressing the same plasmodial antigen. *Proc. Natl. Acad. Sci. U.S.A.* 98 11491–11496 11553779 10.1073/pnas.191380898 PMC58757

[B17] Casey K. A. Fraser K. A. Schenkel J. M. Moran A. Abt M. C. Beura L. K. (2012). Antigen-independent differentiation and maintenance of effector-like resident memory T cells in tissues. *J. Immunol.* 188 4866–4875 22504644 10.4049/jimmunol.1200402 PMC3345065

[B18] Casimiro D. R. Chen L. Fu T. M. Evans R. K. Caulfield M. J. Davies M. E. (2003). Comparative immunogenicity in rhesus monkeys of DNA plasmid, recombinant vaccinia virus, and replication-defective adenovirus vectors expressing a human immunodeficiency virus type 1 gag gene. *J. Virol.* 77 6305–6313 12743287 10.1128/JVI.77.11.6305-6313.2003 PMC154996

[B19] Casimiro D. R. Wang F. Schleif W. A. Liang X. Zhang Z. Q. Tobery T. W. (2005). Attenuation of simian immunodeficiency virus SIVmac239 infection by prophylactic immunization with DNA and recombinant adenoviral vaccine vectors expressing Gag. *J. Virol.* 79 15547–15555 16306625 10.1128/JVI.79.24.15547-15555.2005 PMC1315991

[B20] Cayabyab M. J. Korioth-Schmitz B. Sun Y. Carville A. Balachandran H. Miura A. (2009). Recombinant *Mycobacterium bovis* BCG prime-recombinant adenovirus boost vaccination in rhesus monkeys elicits robust polyfunctional simian immunodeficiency virus-specific T-cell responses. *J. Virol.* 83 5505–5513 19297477 10.1128/JVI.02544-08 PMC2681969

[B21] Cebere I. Dorrell L. McShane H. Simmons A. McCormack S. Schmidt C. (2006). Phase I clinical trial safety of DNA- and modified virus Ankara-vectored human immunodeficiency virus type 1 (HIV-1) vaccines administered alone and in a prime-boost regime to healthy HIV-1-uninfected volunteers. *Vaccine* 24 417–425 16176847 10.1016/j.vaccine.2005.08.041

[B22] Chopera D. R. Wright J. K. Brockman M. A. Brumme Z. L. (2011). Immune-mediated attenuation of HIV-1. *Future Virol.* 6 917–928 22393332 10.2217/fvl.11.68 PMC3292540

[B23] Churchyard G. J. Morgan C. Adams E. Hural J. Graham B. S. Moodie Z. (2011). A phase IIA randomized clinical trial of a multiclade HIV-1 DNA prime followed by a multiclade rAd5 HIV-1 vaccine boost in healthy adults (HVTN204). *PLoS ONE* 6 e21225 10.1371/journal.pone.0021225 PMC3152265 21857901

[B24] Cockburn I. A. Chakravarty S. Overstreet M. G. García-Sastre A. Zavala F. (2008). Memory CD8+ T cell responses expand when antigen presentation overcomes T cell self-regulation. *J. Immunol.* 180 64–71 18097005 10.4049/jimmunol.180.1.64

[B25] Cox K. S. Clair J. H. Prokop M. T. Sykes K. J. Dubey S. A. Shiver J. H. (2008). DNA gag/adenovirus type 5 (Ad5) gag and Ad5 gag/Ad5 gag vaccines induce distinct T-cell response profiles. *J. Virol.* 82 8161–8171 18524823 10.1128/JVI.00620-08 PMC2519591

[B26] Cui W. Kaech S. M. (2010). Generation of effector CD8+ T cells and their conversion to memory T cells. *Immunol. Rev.* 236 151–166 20636815 10.1111/j.1600-065X.2010.00926.x PMC4380273

[B27] de Alencar B. C. G. Persechini P. M. Haolla F. A. De Oliveira G. Silverio J. C. Lannes-Vieira J. (2009). Perforin and gamma interferon expression are required for CD4+ and CD8+ T-Cell-dependent protective immunity against a human parasite, *Trypanosoma cruzi*, elicited by heterologous plasmid DNA prime-recombinant adenovirus 5 boost vaccination. *Infect. Immun.* 77 4383–4395 19651871 10.1128/IAI.01459-08 PMC2747960

[B28] De Rosa S. C. Thomas E. P. Bui J. Huang Y. deCamp A. Morgan C. (2011). National institute of allergy and infectious diseases HIV vaccine trials network. V-DNA priming alters T cell responses to HIV-adenovirus vaccine even when responses to DNA are undetectable. *J. Immunol.* 187 3391–3401 21844392 10.4049/jimmunol.1101421 PMC3180898

[B29] Dominguez M. R. Ersching J. Lemos R. Machado A. V. Bruna-Romero O. Rodrigues M. M. (2012). Re-circulation of lymphocytes mediated by sphingosine-1-phosphate receptor-1 contributes to resistance against experimental infection with the protozoan parasite *Trypanosoma cruzi*. *Vaccine* 30 2882–2891 22381075 10.1016/j.vaccine.2012.02.037

[B30] Dominguez M. R. Silveira E. L. de Vasconcelos J. R. de Alencar B. C. Machado A. V. Bruna-Romero O. (2011). Subdominant/cryptic CD8 T cell epitopes contribute to resistance against experimental infection with a human protozoan parasite. *PLoS ONE* 6 e22011 10.1371/journal.pone.0022011 PMC3136500 21779365

[B31] Dudani R. Murali-Krishna K. Krishnan L. Sad S. (2008). IFN-gamma induces the erosion of preexisting CD8 T cell memory during infection with a heterologous intracellular bacterium. *J. Immunol.* 181 1700–1709 18641306 10.4049/jimmunol.181.3.1700 PMC4015950

[B32] Duke C. M. Maguire C. A. Keefer M. C. Federoff H. J. Bowers W. J. Dewhurst S. (2007). HSV-1 amplicon vectors elicit polyfunctional T cell responses to HIV-1 Env, and strongly boost responses to an adenovirus prime. *Vaccine* 25 7410–7421 17868958 10.1016/j.vaccine.2007.08.015 PMC2092414

[B33] Dunachie S. J. Walther M. Epstein J. E. Keating S. Berthoud T. Andrews L. (2006). DNA prime-modified vaccinia virus Ankara boost vaccine encoding thrombospondin-related adhesion protein but not circumsporozoite protein partially protects healthy malaria-naive adults against *Plasmodium falciparum* sporozoite challenge. *Infect. Immun.* 74 5933–5942 16988273 10.1128/IAI.00590-06 PMC1594937

[B34] Ellenberger D. Otten R. A. Li B. Aidoo M. Rodriguez I. V. Sariol C. A. (2006). HIV-1 DNA/MVA vaccination reduces the per exposure probability of infection during repeated mucosal SHIV challenges. *Virology* 352 216–225 16725169 10.1016/j.virol.2006.04.005

[B35] Elvang T. Christensen J. P. Billeskov R. Thi Kim Thanh Hoang T. Holst P. Thomsen A. R. (2009). CD4 and CD8 T cell responses to the *M. tuberculosis* Ag85B-TB10.4 promoted by adjuvanted subunit, adenovector or heterologous prime boost vaccination. *PLoS ONE* 4 e5139 10.1371/journal.pone.0005139 PMC2663846 19357780

[B36] Finn J. D. Bassett J. Millar J. B. Grinshtein N. Yang T. C. Parsons R. (2009). Persistence of transgene expression influences CD8+ T-cell expansion and maintenance following immunization with recombinant adenovirus. *J. Virol.* 83 12027–12036 19759135 10.1128/JVI.00593-09 PMC2786755

[B37] Flatz L. Roychoudhuri R. Honda M. Filali-Mouhim A. Goulet J. P. Kettaf N. (2011). Single-cell gene-expression profiling reveals qualitatively distinct CD8 T cells elicited by different gene-based vaccines. *Proc. Natl. Acad. Sci. U.S.A.* 108 5724–5729 21422297 10.1073/pnas.1013084108 PMC3078363

[B38] Frahm N. DeCamp A. C. Friedrich D. P. Carter D. K. Defawe O. D. Kublin J. G. (2012). Human adenovirus-specific T cells modulate HIV-specific T cell responses to an Ad5-vectored HIV-1 vaccine. *J. Clin. Invest.* 122 359–367 22201684 10.1172/JCI60202 PMC3248307

[B39] Freel S. A. Lamoreaux L. Chattopadhyay P. K. Saunders K. Zarkowsky D. Overman R. G. (2010). Phenotypic and functional profile of HIV-inhibitory CD8 T cells elicited by natural infection and heterologous prime/boost vaccination. *J. Virol.* 84 4998–5006 20200250 10.1128/JVI.00138-10 PMC2863846

[B40] Gebhardt T. Wakim L. M. Eidsmo L. Reading P. C. Heath W. R. Carbone F. R. (2009). Memory T cells in nonlymphoid tissue that provide enhanced local immunity during infection with herpes simplex virus. *Nat. Immunol.* 10 524–530 19305395 10.1038/ni.1718

[B41] Geisbert T. W. Bailey M. Geisbert J. B. Asiedu C. Roederer M. Grazia-Pau M. (2010). Vector choice determines immunogenicity and potency of genetic vaccines against Angola Marburg virus in nonhuman primates. *J. Virol.* 84 10386–10394 20660192 10.1128/JVI.00594-10 PMC2937810

[B42] Gilbert S. C. Moorthy V. S. Andrews L. Pathan A. A. McConkey S. J. Vuola J. M. (2006). Synergistic DNA-MVA prime-boost vaccination regimes for malaria and tuberculosis. *Vaccine* 24 4554–4561 16150517 10.1016/j.vaccine.2005.08.048

[B43] Gilbert S. C. Schneider J. Hannan C. M. Hu J. T. Plebanski M. Sinden R. (2002). Enhanced CD8 T cell immunogenicity and protective efficacy in a mouse malaria model using a recombinant adenoviral vaccine in heterologous prime-boost immunisation regimes. *Vaccine* 20 1039–1045 11803063 10.1016/s0264-410x(01)00450-9

[B44] Gómez C. E. Nájera J. L. Krupa M. Perdiguero B. Esteban M. (2011). MVA and NYVAC as vaccines against emergent infectious diseases and cancer. *Curr. Gene Ther.* 11 189–217 21453284 10.2174/156652311795684731

[B45] Goonetilleke N. Moore S. Dally L. Winstone N. Cebere I. Mahmoud A. (2006). Induction of multifunctional human immunodeficiency virus type 1 (HIV-1)-specific T cells capable of proliferation in healthy subjects by using a prime-boost regimen of DNA- and modified vaccinia virus Ankara-vectored vaccines expressing HIV-1 Gag coupled to CD8+ T-cell epitopes. *J. Virol.* 80 4717–4728 16641265 10.1128/JVI.80.10.4717-4728.2006 PMC1472051

[B46] Green D. R. (2008). Fas Bim boom! *Immunity* 28 141–143 18275825 10.1016/j.immuni.2008.01.004

[B47] Hansen S. G. Ford J. C. Lewis M. S. Ventura A. B. Hughes C. M. Coyne-Johnson L. (2011). Profound early control of highly pathogenic SIV by an effector memory T-cell vaccine. *Nature* 473 523–527 21562493 10.1038/nature10003 PMC3102768

[B48] Harari A. Bart P. A. Stöhr W. Tapia G. Garcia M. Medjitna-Rais E. (2008). An HIV-1 clade C DNA prime, NYVAC boost vaccine regimen induces reliable, polyfunctional, and long-lasting T cell responses. *J. Exp. Med.* 205 63–77 18195071 10.1084/jem.20071331 PMC2234371

[B49] Haut L. H. Ratcliffe S. Pinto A. R. Ertl H. (2011). Effect of preexisting immunity to adenovirus on transgene product-specific genital T cell responses on vaccination of mice with a homologous vector. *J. Infect. Dis.* 203 1073–1081 21450997 10.1093/infdis/jiq161 PMC3107557

[B50] Helbig C. Gentek R. Backer R. A. de Souza Y. Derks I. A. Eldering E. (2012). Notch controls the magnitude of T helper cell responses by promoting cellular longevity. *Proc. Natl. Acad. Sci. U.S.A.* 109 9041–9046 22615412 10.1073/pnas.1206044109 PMC3384214

[B51] Hensley L. E. Mulangu S. Asiedu C. Johnson J. Honko A. N. Stanley D. (2010). Demonstration of cross-protective vaccine immunity against an emerging pathogenic Ebolavirus species. *PLoS Pathog.* 6 e1000904 10.1371/journal.ppat.1000904 PMC2873919 20502688

[B52] Hikono H. Kohlmeier J. E. Takamura S. Wittmer S. T. Roberts A. D. Woodland D. L. (2007). Activation phenotype, rather than central- or effector-memory phenotype, predicts the recall efficacy of memory CD8+ T cells. *J. Exp. Med.* 204 1625–1636 17606632 10.1084/jem.20070322 PMC2118640

[B53] Hoft D. F. Blazevic A. Stanley J. Landry B. Sizemore D. Kpamegan E. (2012). A recombinant adenovirus expressing immunodominant TB antigens can significantly enhance BCG-induced human immunity. *Vaccine* 30 2098–2108 22296955 10.1016/j.vaccine.2012.01.048

[B54] Hughes P. D. Belz G. T. Fortner K. A. Budd R. C. Strasser A. Bouillet P. (2008). Apoptosis regulators Fas and Bim cooperate in shutdown of chronic immune responses and prevention of autoimmunity. *Immunity* 28 197–205 18275830 10.1016/j.immuni.2007.12.017 PMC2270348

[B55] Huster K. M. Koffler M. Stemberger C. Schiemann M. Wagner H. Busch D. H. (2006). Unidirectional development of CD8+ central memory T cells into protective Listeria-specific effector memory T cells. *Eur. J. Immunol.* 36 1453–1464 16637009 10.1002/eji.200635874

[B56] Huster K. M. Stemberger C. Gasteiger G. Kastenmüller W. Drexler I. Busch D. H. (2009). Cutting edge: memory CD8 T cell compartment grows in size with immunological experience but nevertheless can lose function. *J. Immunol.* 183 6898–6902 19890045 10.4049/jimmunol.0902454

[B57] Hutnick N. A. Myles D. J. Hirao L. Scott V. L. Ferraro B. Khan A. S. (2012). An optimized SIV DNA vaccine can serve as a boost for Ad5 and provide partial protection from a high-dose SIVmac251 challenge. *Vaccine* 30 3202–3208 22406458 10.1016/j.vaccine.2012.02.069

[B58] Irvine K. R. Chamberlain R. S. Shulman E. P. Surman D. R. Rosenberg S. A. Restifo N. P. (1997). Enhancing efficacy of recombinant anticancer vaccines with prime/boost regimens that use two different vectors. *J. Natl. Cancer Inst.* 89 1595–1601 9362157 10.1093/jnci/89.21.1595

[B59] Jameson S. C. Masopust D. (2009). Diversity in T cell memory: an embarrassment of riches. *Immunity* 31 859–871 20064446 10.1016/j.immuni.2009.11.007 PMC2957815

[B60] Jaoko W. Karita E. Kayitenkore K. Omosa-Manyonyi G. Allen S. Than S. (2010). Safety and immunogenicity study of multiclade HIV-1 adenoviral vector vaccine alone or as boost following a multiclade HIV-1 DNA vaccine in Africa. *PLoS ONE* 5 e12873 10.1371/journal.pone.0012873 PMC2943475 20877623

[B61] Jiang X. Clark R. A. Liu L. Wagers A. J. Fuhlbrigge R. C. Kupper T. S. (2012). Skin infection generates non-migratory memory CD8+ T(RM) cells providing global skin immunity. *Nature* 483 227–231 22388819 10.1038/nature10851 PMC3437663

[B62] Kaufman D. R. Bivas-Benita M. Simmons N. L. Miller D. Barouch D. H. (2010). Route of adenovirus-based HIV-1 vaccine delivery impacts the phenotype and trafficking of vaccine-elicited CD8+ T lymphocytes. *J. Virol.* 84 5986–5996 20357087 10.1128/JVI.02563-09 PMC2876628

[B63] Keating S. M. Bejon P. Berthoud T. Vuola J. M. Todryk S. Webster D. P. (2005). Durable human memory T cells quantifiable by cultured enzyme-linked immunospot assays are induced by heterologous prime boost immunization and correlate with protection against malaria. *J. Immunol.* 175 5675–5680 16237057 10.4049/jimmunol.175.9.5675

[B64] Kohlmeier J. E. Reiley W. W. Perona-Wright G. Freeman M. L. Yager E. J. Connor L. M. (2011). Inflammatory chemokine receptors regulate CD8+ T cell contraction and memory generation following infection. *J. Exp. Med.* 208 1621–1634 21788409 10.1084/jem.20102110 PMC3149221

[B65] Koup R. A. Roederer M. Lamoreaux L. Fischer J. Novik L. Nason M. C. (2010). Priming immunization with DNA augments immunogenicity of recombinant adenoviral vectors for both HIV-1 specific antibody and T-cell responses. *PLoS ONE* 5 e9015 10.1371/journal.pone.0009015 PMC2814848 20126394

[B66] Kursar M. Jnner N. Pfeffer K. Brinkmann V. Kaufmann S. H Mittrücker H. W. (2008). Requirement of secondary lymphoid tissues for the induction of primary and secondary T cell responses against *Listeria monocytogenes*. *Eur. J. Immunol.* 38 127–138 18050270 10.1002/eji.200737142

[B67] Lai L. Kwa S. Kozlowski P. A. Montefiori D. C. Ferrari G. Johnson W. E. (2011). Prevention of infection by a granulocyte-macrophage colony-stimulating factor co-expressing DNA/modified vaccinia Ankara simian immunodeficiency virus vaccine. *J. Infect. Dis.* 204 164–173 21628671 10.1093/infdis/jir199 PMC3143670

[B68] Lakhashe S. K. Velu V. Sciaranghella G. Siddappa N. B. Dipasquale J. M. Hemashettar G. (2011). Prime-boost vaccination with heterologous live vectors encoding SIV gag and multimeric HIV-1 gp160 protein: efficacy against repeated mucosal R5 clade C SHIV challenges. *Vaccine* 29 5611–5622 21693155 10.1016/j.vaccine.2011.06.017 PMC3154722

[B69] Lasaro M. O. Ertl H. C. (2009). New insights on adenovirus as vaccine vectors. *Mol. Ther.* 17 1333–1339 19513019 10.1038/mt.2009.130 PMC2835230

[B70] Letvin N. L. Mascola J. R. Sun Y. Gorgone D. A. Buzby A. P. Xu L. (2006). Preserved CD4+ central memory T cells and survival in vaccinated SIV-challenged monkeys. *Science* 312 1530–1533 16763152 10.1126/science.1124226 PMC2365913

[B71] Li Q. Rao R. Vazzana J. Goedegebuure P. Odunsi K. Gillanders W. (2012). Regulating mammalian target of rapamycin to tune vaccination-induced CD8+ T cell responses for tumor immunity. *J. Immunol.* 188 3080–3087 22379028 10.4049/jimmunol.1103365 PMC3311730

[B72] Li S. Rodrigues M. Rodriguez D. Rodriguez J. R. Esteban M. Palese P. (1993). Priming with recombinant influenza virus followed by administration of recombinant vaccinia virus induces CD8+ T-cell-mediated protective immunity against malaria. *Proc. Natl. Acad. Sci. U.S.A.* 90 5214–5218 7685119 10.1073/pnas.90.11.5214 PMC46686

[B73] Liu J. Ewald B. A. Lynch D. M. Denholtz M. Abbink P. Lemckert A. A. (2008). Magnitude and phenotype of cellular immune responses elicited by recombinant adenovirus vectors and heterologous prime-boost regimens in rhesus monkeys. *J. Virol.* 82 4844–4852 18337575 10.1128/JVI.02616-07 PMC2346755

[B74] Mackay L. K. Stock A. T. Ma J. Z. Jones C. M. Kent S. J. Mueller S. N. (2012). Long-lived epithelial immunity by tissue-resident memory T (TRM) cells in the absence of persisting local antigen presentation. *Proc. Natl. Acad. Sci. U.S.A.* 109 7037–7042 22509047 10.1073/pnas.1202288109 PMC3344960

[B75] Magalhaes I. Sizemore D. R. Ahmed R. K. Mueller S. Wehlin L. Scanga C. (2008). rBCG induces strong antigen-specific T cell responses in rhesus macaques in a prime-boost setting with an adenovirus 35 tuberculosis vaccine vector. *PLoS ONE* 3 e3790 10.1371/journal.pone.0003790 PMC2582491 19023426

[B76] Martinon F. Brochard P. Ripaux M. Delache B. Aurégan G. Vaslin B. (2008). Improved protection against simian immunodeficiency virus mucosal challenge in macaques primed with a DNA vaccine and boosted with the recombinant modified vaccinia virus Ankara and recombinant Semliki Forest virus. *Vaccine* 26 532–545 18093703 10.1016/j.vaccine.2007.11.025

[B77] Martins M. A. Wilson N. A. Reed J. S. Ahn C. D. Klimentidis Y. C. Allison D. B. (2010). T-cell correlates of vaccine efficacy after a heterologous simian immunodeficiency virus challenge. *J. Virol.* 84 4352–4365 20164222 10.1128/JVI.02365-09 PMC2863752

[B78] Masopust D. Choo D. Vezys V. Wherry E. J. Duraiswamy J. Akondy R. (2010). Dynamic T cell migration program provides resident memory within intestinal epithelium. *J. Exp. Med.* 207 553–564 20156972 10.1084/jem.20090858 PMC2839151

[B79] Masopust D. Ha S. J. Vezys V. Ahmed R. (2006). Stimulation history dictates memory CD8 T cell phenotype: implications for prime-boost vaccination. *J. Immunol.* 177 831–839 16818737 10.4049/jimmunol.177.2.831

[B80] Mattapallil J. J. Douek D. C. Buckler-White A. Montefiori D. Letvin N. L. Nabel G. J. (2006). Vaccination preserves CD4 memory T cells during acute simian immunodeficiency virus challenge. *J. Exp. Med.* 203 1533–1541 16735692 10.1084/jem.20060657 PMC2118314

[B81] McConkey S. J. Reece W. H. Moorthy V. S. Webster D. Dunachie S. Butcher G. (2003). Enhanced T-cell immunogenicity of plasmid DNA vaccines boosted by recombinant modified vaccinia virus Ankara in humans. *Nat. Med.* 9 729–735 12766765 10.1038/nm881

[B82] McShane H. Brookes R. Gilbert S. C. Hill A. V. (2001). Enhanced immunogenicity of CD4+ T-cell responses and protective efficacy of a DNA-modified vaccinia virus Ankara prime-boost vaccination regimen for murine tuberculosis. *Infect. Immun.* 69 681–686 11159955 10.1128/IAI.69.2.681-686.2001 PMC97939

[B83] Moore A. C. Hill A. V. (2004). Progress in DNA-based heterologous prime-boost immunization strategies for malaria. *Immunol. Rev.* 199 126–143 15233731 10.1111/j.0105-2896.2004.00138.x

[B84] Moorthy V. S. Imoukhuede E. B. Milligan P. Bojang K. Keating S. Kaye P. (2004). A randomised, double-blind, controlled vaccine efficacy trial of DNA/MVA ME-TRAP against malaria infection in Gambian adults. *PLoS Med.* 1 e33 10.1371/journal.pmed.0010033 PMC524376 15526058

[B85] Mudd P. A. Watkins D. I. (2011). Understanding animal models of elite control: windows on effective immune responses against immunodeficiency viruses. *Curr. Opin. HIV AIDS* 6 197–201 21502922 10.1097/COH.0b013e3283453e16 PMC3789597

[B86] Murata K. García-Sastre A. Tsuji M. Rodrigues M. Rodriguez D. Rodriguez J. R. (1996). Characterization of in vivo primary and secondary CD8+ T cell responses induced by recombinant influenza and vaccinia viruses. *Cell. Immunol.* 173 96–107 8871605 10.1006/cimm.1996.0255

[B87] Nigam P. Earl P. L. Americo J. L. Sharma S. Wyatt L. S. Edghill-Spano Y. (2007). DNA/MVA HIV-1/AIDS vaccine elicits long-lived vaccinia virus-specific immunity and confers protection against a lethal monkeypox challenge. *Virology* 151 73–83 17507071 10.1016/j.virol.2007.04.010 PMC2072046

[B88] Nolz J. C. Harty J. T. (2011). Strategies and implications for prime-boost vaccination to generate memory CD8 T cells. *Adv. Exp. Med. Biol.* 780 69–83 21842366 10.1007/978-1-4419-5632-3_7

[B89] Peiperl L. Morgan C. Moodie Z. Li H. Russell N. Graham B. S. (2010). Safety and immunogenicity of a replication-defective adenovirus type 5 HIV vaccine in Ad5-seronegative persons: a randomized clinical trial (HVTN 054). *PLoS ONE* 5 e13579 10.1371/journal.pone.0013579 PMC2965084 21048953

[B90] Pinschewer D. D. Ochsenbein A. F. Odermatt B. Brinkmann V. Hengartner H. Zinkernagel R. M. (2000). FTY720 immunosuppression impairs effector T cell peripheral homing without affecting induction, expansion, and memory. *J. Immunol.* 164 5761–5770 10820254 10.4049/jimmunol.164.11.5761

[B91] Pinto A. R. Fitzgerald J. C. Giles-Davis W. Gao G. P. Wilson J. M. Ertl H. C. (2003). Induction of CD8+ T cells to an HIV-1 antigen through a prime boost regimen with heterologous E1-deleted adenoviral vaccine carriers. *J. Immunol.* 171 6774–6779 14662882 10.4049/jimmunol.171.12.6774

[B92] Porter D. W. Thompson F. M. Berthoud T. K. Hutchings C. L. Andrews L. Biswas S. (2011). A human phase I/IIa malaria challenge trial of a polyprotein malaria vaccine. *Vaccine* 29 7514–7522 21501642 10.1016/j.vaccine.2011.03.083 PMC3195259

[B93] Pulko V. Harris K. J. Liu X. Gibbons R. M. Harrington S. M. Krco C. J. (2011). B7-h1 expressed by activated CD8 T cells is essential for their survival. *J. Immunol.* 187 5606–5614 22025548 10.4049/jimmunol.1003976 PMC3221917

[B94] Purwar R. Campbell J. Murphy G. Richards W. G. Clark R. A. Kupper T. S. (2011). Resident memory T cells (T(RM)) are abundant in human lung: diversity, function, and antigen specificity. *PLoS ONE* 6 e16245 10.1371/journal.pone.0016245 PMC3027667 21298112

[B95] Reyes-Sandoval A. Berthoud T. Alder N. Siani L. Gilbert S. C. Nicosia A. (2010). Prime-boost immunization with adenoviral and modified vaccinia virus Ankara vectors enhances the durability and polyfunctionality of protective malaria CD8+ T-cell responses. *Infect. Immun.* 78 145–153 19858306 10.1128/IAI.00740-09 PMC2798185

[B96] Reyes-Sandoval A. Wyllie D. H. Bauza K. Milicic A. Forbes E. K. Rollier C. S. (2011). CD8+ T effector memory cells protect against liver-stage malaria. *J. Immunol.* 187 1347–1357 21715686 10.4049/jimmunol.1100302 PMC4568294

[B97] Rice J. Ottensmeier C. H. Stevenson F. K. (2008). DNA vaccines: precision tools for activating effective immunity against cancer. *Nat. Rev. Cancer* 8 108–120 18219306 10.1038/nrc2326

[B98] Rigato P. O. De Alencar B. C. De Vasconcelos J. R. C. Dominguez M. R. Araujo A. F. Machado A. V. (2011). Heterologous plasmid DNA prime-recombinant human adenovirus 5 boost vaccination generates a stable pool of protective long-lived CD8+ T effector memory cells specific for a human parasite, *Trypanosoma cruzi*. *Infect. Immun.* 79 2120–2130 21357719 10.1128/IAI.01190-10 PMC3088135

[B99] Rodríguez D. González-Aseguinolaza G. Rodríguez J. R. Vijayan A. Gherardi M. Rueda P. (2012). Vaccine efficacy against malaria by the combination of porcine parvovirus-like particles and vaccinia virus vectors expressing CS of *Plasmodium*. *PLoS ONE* 7 e34445 10.1371/journal.pone.0034445 PMC3328484 22529915

[B100] Rodrigues M. Li S. Murata K. Rodriguez D. Rodriguez J. R. Bacik I. (1994). Influenza and vaccinia viruses expressing malaria CD8+ T and B cell epitopes. Comparison of their immunogenicity and capacity to induce protective immunity. *J. Immunol.* 153 4636–4648 7525709

[B101] Saade F. Petrovsky N. (2012). Technologies for enhanced efficacy of DNA vaccines. *Expert Rev. Vaccines* 11 189–209 22309668 10.1586/erv.11.188 PMC3293989

[B102] Sadagopal S. Amara R. R. Kannanganat S. Sharma S. Chennareddi L. Robinson H. L. (2008). Expansion and exhaustion of T-cell responses during mutational escape from long-term viral control in two DNA/modified vaccinia virus Ankara-vaccinated and simian-human immunodeficiency virus SHIV-89.6P-challenged macaques. *J. Virol.* 82 4149–4153 18234787 10.1128/JVI.02242-07 PMC2292989

[B103] Santra S. Seaman M. S. Xu L. Barouch D. H. Lord C. I. Lifton M. A. (2005). Replication-defective adenovirus serotype 5 vectors elicit durable cellular and humoral immune responses in nonhuman primates. *J. Virol.* 79 6516–6522 15858035 10.1128/JVI.79.10.6516-6522.2005 PMC1091731

[B104] Schirmbeck R. Reimann J. Kochanek S. Kreppel F. (2008). The immunogenicity of adenovirus vectors limits the multispecificity of CD8 T-cell responses to vector-encoded transgenic antigens. *Mol. Ther.* 16 1609–1616 18612271 10.1038/mt.2008.141

[B105] Schmidt N. W. Harty J. T. (2011). Cutting edge: attrition of *Plasmodium*-specific memory CD8 T cells results in decreased protection that is rescued by booster immunization. *J. Immunol.* 186 3836–3840 21357257 10.4049/jimmunol.1003949 PMC3074438

[B106] Schneider J. Gilbert S. C. Blanchard T. J. Hanke T. Robson K. J. Hannan C. M. (1998). Enhanced immunogenicity for CD8 T cell induction and complete protective efficacy of malaria DNA vaccination by boosting with modified vaccinia virus Ankara. *Nat. Med.* 4 397–402 9546783 10.1038/nm0498-397

[B107] Schooley R. T. Spritzler J. Wang H. Lederman M. M. Havlir D. Kuritzkes D. R. (2010). AIDS clinical trials group 5197: a placebo-controlled trial of immunization of HIV-1-infected persons with a replication-deficient adenovirus type 5 vaccine expressing the HIV-1 core protein. *J. Infect. Dis.* 202 705–716 20662716 10.1086/655468 PMC2916952

[B108] Sedegah M. Jones T. R. Kaur M. Hedstrom R. Hobart P. Tine J. A. (1998). Boosting with recombinant vaccinia increases immunogenicity and protective efficacy of malaria DNA vaccine. *Proc. Natl. Acad. Sci. U.S.A.* 95 7648–7653 9636204 10.1073/pnas.95.13.7648 PMC22711

[B109] Selin L. K. Lin M. Y. Kraemer K. A. Pardoll D. M. Schneck J. P. Varga S. M. (1999). Attrition of T cell memory: selective loss of LCMV epitope-specific memory CD8 T cells following infections with heterologous viruses. *Immunity* 11 733–742 10626895 10.1016/s1074-7613(00)80147-8

[B110] Selin L. K. Vergilis K. Welsh R. M. Nahill S. R. (1996). Reduction of otherwise remarkably stable virus-specific cytotoxic T lymphocyte memory by heterologous viral infections. *J. Exp. Med.* 183 2489–2499 8676069 10.1084/jem.183.6.2489 PMC2192604

[B111] Sheridan B. S. Lefrançois L. (2011). Regional and mucosal memory T cells. *Nat. Immunol.* 12 485–491 21739671 10.1038/ni.2029 PMC3224372

[B112] Streeck H. Nixon D. F. (2010). T cell immunity in acute HIV-1 infection. *J. Infect. Dis.* 202(Suppl. 2) S302–S308 20846037 10.1086/655652 PMC2954287

[B113] Sun Y. Schmitz J. E. Buzby A. P. Barker B. R. Rao S. S. Xu L. (2006). Virus-specific cellular immune correlates of survival in vaccinated monkeys after simian immunodeficiency virus challenge. *J. Virol.* 80 10950–10956 16943292 10.1128/JVI.01458-06 PMC1642180

[B114] Suthar M. S. Ramos H. J. Brassil M. M. Netland J. Chappell C. P. Blahnik G. (2012). The RIG-I-like receptor LGP2 controls CD8+ T cell survival and fitness. *Immunity* 37 235–248 22841161 10.1016/j.immuni.2012.07.004 PMC3910444

[B115] Takai S. Sabzevari H. Farsaci B. Schlom J. Greiner J. W. (2012). Distinct effects of saracatinib on memory CD8+ T cell differentiation. *J. Immunol.* 188 4323–4333 22450814 10.4049/jimmunol.1101439 PMC3378668

[B116] Tao D. Barba-Spaeth G. Rai U. Nussenzweig V. Rice C. M. Nussenzweig R. S. (2005). Yellow fever 17D as a vaccine vector for microbial CTL epitopes: protection in a rodent malaria model. *J. Exp. Med.* 201 201–209 15657290 10.1084/jem.20041526 PMC2212788

[B117] Tatsis N. Fitzgerald J. C. Reyes-Sandoval A. Harris-McCoy K. C. Hensley S. E. Zhou D. (2007). Adenoviral vectors persist in vivo and maintain activated CD8+ T cells: implications for their use as vaccines. *Blood* 110 1916–1923 17510320 10.1182/blood-2007-02-062117 PMC1976365

[B118] Vasconcelos J. R. Bruña-Romero O. Araújo A. F. Dominguez M. R. Ersching J. de Alencar B. C. (2012). Pathogen-induced proapoptotic phenotype and high CD95 (Fas) expression accompany a suboptimal CD8+ T-cell response: reversal by adenoviral vaccine. *PLoS Pathog.* 8 e1002699 10.1371/journal.ppat.1002699 PMC3355083 22615561

[B119] Vezys V. Yates A. Casey K. A. Lanier G. Ahmed R. Antia R. (2009). Memory CD8 T-cell compartment grows in size with immunological experience. *Nature* 457 196–199 19005468 10.1038/nature07486

[B120] Vijayan A. Gómez C. E. Espinosa D. A. Goodman A. G. Sanchez-Sampedro L. Sorzano C. O. (2012). Adjuvant-like effect of vaccinia virus 14K protein: a case study with malaria vaccine based on the circumsporozoite protein. *J. Immunol.* 188 6407–6417 22615208 10.4049/jimmunol.1102492 PMC4181723

[B121] Vojnov L. Bean A. T. Peterson E. J. Chiuchiolo M. J. Sacha J. B. Denes F. S. (2011). DNA/Ad5 vaccination with SIV epitopes induced epitope-specific CD4+ T cells, but few subdominant epitope-specific CD8+ T cells. *Vaccine* 29 7483–7490 21839132 10.1016/j.vaccine.2011.07.048 PMC3186839

[B122] Vuola J. M. Keating S. Webster D. P. Berthoud T. Dunachie S. Gilbert S. C. (2005). Differential immunogenicity of various heterologous prime-boost vaccine regimens using DNA and viral vectors in healthy volunteers. *J. Immunol.* 174 449–455 15611270 10.4049/jimmunol.174.1.449

[B123] Wakim L. M. Waithman J. van Rooijen N. Heath W. R. Carbone F. R. (2008). Dendritic cell-induced memory T cell activation in nonlymphoid tissues. *Science* 319 198–202 18187654 10.1126/science.1151869

[B124] Wakim L. M. Woodward-Davis A. Bevan M. J. (2010). Memory T cells persisting within the brain after local infection show functional adaptations to their tissue of residence. *Proc. Natl. Acad. Sci. U.S.A.* 107 17872–17879 20923878 10.1073/pnas.1010201107 PMC2964240

[B125] Wakim L. M. Woodward-Davis A. Liu R. Hu Y. Villadangos J. Smyth G. (2012). The molecular signature of tissue resident memory CD8 T cells isolated from the brain. *J. Immunol.* 189 3462–3471 22922816 10.4049/jimmunol.1201305 PMC3884813

[B126] Weant A. E. Michalek R. D. Khan I. U. Holbrook B. C. Willingham M. C. Grayson J. M. (2008). Apoptosis regulators Bim and Fas function concurrently to control autoimmunity and CD8+ T cell contraction. *Immunity* 28 218–230 18275832 10.1016/j.immuni.2007.12.014

[B127] Wiesel M. Walton S. Richter K. Oxenius A. (2009). Virus-specific CD8 T cells: activation, differentiation and memory formation. *APMIS* 117 356–381 19400862 10.1111/j.1600-0463.2009.02459.x

[B128] Wilks A. B. Christian E. C. Seaman M. S. Sircar P. Carville A. Gomez C. E. (2010). Robust vaccine-elicited cellular immune responses in breast milk following systemic simian immunodeficiency virus DNA prime and live virus vector boost vaccination of lactating rhesus monkeys. *J. Immunol.* 185 7097–7106 21041730 10.4049/jimmunol.1002751 PMC3017679

[B129] Wilson N. A. Reed J. Napoe G. S. Piaskowski S. Szymanski A. Furlott J. (2006). Vaccine-induced cellular immune responses reduce plasma viral concentrations after repeated low-dose challenge with pathogenic simian immunodeficiency virus SIVmac239. *J. Virol.* 80 5875–5885 16731926 10.1128/JVI.00171-06 PMC1472612

[B130] Winstone N. Guimarães-Walker A. Roberts J. Brown D. Loach V. Goonetilleke N. (2009). Increased detection of proliferating, polyfunctional, HIV-1-specific T cells in DNA-modified vaccinia virus Ankara-vaccinated human volunteers by cultured IFN-gamma ELISPOT assay. *Eur. J. Immunol.* 39 975–985 19266489 10.1002/eji.200839167

[B131] Winstone N. Wilson A. J. Morrow G. Boggiano C. Chiuchiolo M. J. Lopez M. (2011). Enhanced control of pathogenic simian immunodeficiency virus SIVmac239 replication in macaques immunized with an interleukin-12 plasmid and a DNA prime-viral vector boost vaccine regimen. *J. Virol.* 85 9578–9587 21734035 10.1128/JVI.05060-11 PMC3165762

[B132] Xiao H. Peng Y. Hong Y. Liu Y. Guo Z. S. Bartlett D. L. (2011). Lentivector prime and vaccinia virus vector boost generate high-quality CD8 memory T cells and prevent autochthonous mouse melanoma. *J. Immunol.* 187 1788–1796 21746967 10.4049/jimmunol.1101138 PMC3150273

[B133] Yamamoto T. Johnson M. J. Price D. A. Wolinsky D. I. Almeida J. R. Petrovas C. (2012). Virus inhibition activity of effector memory CD8+ T cells determines simian immunodeficiency virus load in vaccinated monkeys after vaccine breakthrough infection. *J. Virol.* 86 5877–5884 22419810 10.1128/JVI.00315-12 PMC3347297

[B134] Zavala F. Rodrigues M. Rodriguez D. Rodriguez J. R. Nussenzweig R. S. Esteban M. (2001). A striking property of recombinant poxviruses: efficient inducers of in vivo expansion of primed CD8+ T cells. *Virology* 280 155–159 11162829 10.1006/viro.2000.0792

